# Management and Outcome of Hindfoot Trauma With Concomitant Talar Head Injury

**DOI:** 10.1177/1071100720980023

**Published:** 2021-01-21

**Authors:** Esmee Wilhelmina Maria Engelmann, Olivier Wijers, Jelle Posthuma, Tim Schepers

**Affiliations:** 1 Trauma Unit, Amsterdam University Medical Center, location AMC, Amsterdam, The Netherlands

**Keywords:** talus, talonavicular, Chopart, foot fractures, trauma

## Abstract

**Background:**

Talar head fractures account for 2.6% to 10% of all talar fractures and are often associated with concomitant musculoskeletal injuries. The current literature only describes a total of 14 patients with talar head fractures and, with that, guidelines for management are lacking. The aim of the current study was to evaluate the management and long-term outcome of patients who have hindfoot trauma with concomitant talar head fractures.

**Methods:**

This study includes a retrospective cohort of patients with talar head fractures. Patient characteristics, trauma mechanism, fracture characteristics, treatment, follow-up, and complications were reported. Functional outcome was assessed using the Foot Function Index (FFI) and the American Orthopaedic Foot & Ankle Society (AOFAS) hindfoot score. Quality of life was measured by the EuroQol-5D (EQ-5D). Twenty-one patients with acute fractures of the talar head were identified. The mean follow-up time was 4.9 years.

**Results:**

All patients sustained additional ipsilateral foot and/or ankle injuries. Fifteen patients had operative management of their talar head fracture. There were no postoperative wound infections and no cases of avascular necrosis. All fractures united, and 29% of patients developed posttraumatic osteoarthritis. The overall mean FFI score index was 34.2, and the mean AOFAS score was 70.7. The mean EQ-5D index score was 0.74.

**Conclusion:**

Talar head fractures always coincided with other (foot) fractures. Management and long-term functional outcome were affected by the extent of associated injuries. Due to the low incidence and high complexity of talar head fractures, early referral to dedicated foot surgeons and centralization of complex foot surgery is recommended.

**Level of Evidence:**

Level IV, retrospective case series.

Talar fractures account for less than 1% of all fractures. Even more uncommon are fractures of the talar head, representing just 2.6% to 10% of all talar fractures.^5,10^ This rare type of talar fracture was first described by Coltart^4^ in 1952. Since its identification and description, only 14 cases of talar head fractures have been described in the literature (Table 1). Of those 14, only 5 cases were described as being isolated injuries to the talar head.^2,9^ With this small sample size, demographics and treatment recommendations are lacking.

**Table 1.** Previous Publications on Acute Talar Head Fractures.

Author	Year	n	Mean age, y	Mechanism	Injury pattern	Treatment	Functional outcome	Complications	FU, mo
Mulligan^14^	1986	1	27	Gymnastics	Head and neck^a^ Nondisplaced	Nonoperative	No pain 100% FROM	—	21
Pehlivan^15^	2002	1	22	Walking (inversion)	Medial subtalar dislocation	ORIF (K-wire)	Mild pain 75% ROM	—	26
Vlahovich et al^22^	2005	1	33	Snowboarding	Subtalar impaction Nondisplaced	ORIF (screws)	Pain + swelling in prolonged activity	—	3
Matsumura et al^11^	2008	1	26	Wakeboarding	Impaction, displaced medial head and navicular^a^	Osteotomy, bone graft, screw fixation	No pain 100% ROM	—	12
Ibrahim et al^7^	2015	1	31	Stuck in mud (inversion)	Impaction Locked TNJ^a^ dislocation	ORIF (TNJ bridge plate)	No pain 100% ROM	—	3
Kitamura et al^9^	2019	1	30	Baseball	Isolated Impaction, displacement STJ	ORIF (screws)	No pain 100% ROM	—	65
Anderson^2^	2018	8	31	MVA (6) Height (1) Wakeboarding (1)	Isolated (4) Dislocation^a^ (2)	ORIF (7, lag screws), fragment excision (1)	PROMIS PF mean 42.95; PI 54.57; D 50.84, VAS 2.1/10	—	14.5

Abbreviations: D, depression score; FROM, full range of motion; FU, follow-up; MVA, motor vehicle accident; ORIF, open reduction internal fixation; PF, physical function; PI, Pain Interference score; PROMIS, Patient-Reported Outcomes Measurement Information System; ROM, range of motion; STJ, subtalar joint; TNJ, talonavicular joint; VAS, visual analog scale; —, no complications.

a Fracture.

Trauma to the second largest tarsal bone is associated with challenging injury patterns. This may be largely due to the fact that the majority of the talus is covered with articular cartilage.^12,13^ Fractures of the talus usually occur due to high-energy injuries and are often seen with concomitant musculoskeletal injuries.^6^ The talus has a unique anatomy consisting of a body, neck, and head. The head is convex and articulates with the navicular anteriorly and calcaneus inferiorly, constituting the talonavicular and anterior subtalar joints.^1,20,21^ Its vascular supply is mainly extraosseous, and there is a lack of tendon and muscle attachments.^12,13^ Fractures of the talar head are often associated with peritalar fractures or dislocation.^4,5,7,9,10^ The talar head is part of the medial column of the foot along with the navicular, cuneiforms, and first through third metatarsals. The stability of the medial column is crucial for maintenance of the longitudinal foot arch. Acute talar head fractures may involve the talonavicular junction of the Chopart joint that, along with the subtalar and calcaneocuboid joints, controls hindfoot motion.^2^ Fractures of the talar head, particularly when nondisplaced, are difficult to diagnose and frequently missed on radiographs.^7,11,14^

Given that the talus plays a crucial part in midfoot stability, timely recognition and adequate management of talar head fractures are important for long-term functional outcome of the foot. Positive prognostic factors include early anatomical reduction and articular surface congruency.^9^ Joint misalignment may lead to subtalar or talonavicular posttraumatic arthritis, resulting in instability and chronic pain, causing limitations in daily life.

The aim of the current study was to evaluate the management and long-term outcome of patients who had hindfoot trauma with concomitant talar head fractures treated in a level 1 trauma center.

## Methods

### Design and Patients

A retrospective cohort of all trauma patients with talar head fractures who were treated at our level 1 trauma center between January 1, 2001, and July 1, 2019, was analyzed. Institutional review board approval and informed consent were obtained. Talar head fractures were defined as fractures of the talus involving the articular surface at the talonavicular articulation. Patients younger than 18 years at the day of trauma or with less than 6 months of follow-up time were excluded. Patient-related, clinical, and radiographic data were extracted from the electronic hospital database and picture archiving and communication system (PACS). All patients were evaluated at the outpatient clinic.

### Variables

Variables included age at injury, sex, mechanism of trauma, and concomitant ipsi- or contralateral lower extremity injuries. Imaging was reviewed to categorize the fracture based on anatomical side, articular involvement, joint dislocation, and complexity. Data were collected on the type of treatment, follow-up, complications, and the possible need for implant removal. Complications were defined as postoperative wound infections, avascular necrosis (AVN), nonunion, chronic pain, posttraumatic arthritis, and secondary arthrodesis. Complications specific to talar head injuries were talonavicular osteoarthritis ± fusion and wound complications of the incision used to address the talar head injury. Functional outcome was assessed using the Foot Function Index (FFI; best score 0 points) and the American Orthopaedic Foot & Ankle Society (AOFAS) hindfoot score (best score 100 points). The AOFAS score was divided into groups according to the literature: a score of 90 to 100 was graded as an excellent result, 75 to 89 as good, 50 to 74 as fair, and less than 49 points was graded as a failure or poor outcome. Quality of life (QOL) was measured by the EuroQol-5D (EQ-5D). This included assessment of perceived general health on a visual analog scale (VAS) of 0 to 100, in which 100 represented excellent general health (EQ-VAS). Patient satisfaction was also measured using the VAS of 0 to 10, in which 10 represents the best possible satisfaction.

### Statistical Analysis

The statistical analysis was performed using the Statistical Package for the Social Sciences (SPSS) version 24 (SPSS, Inc). Numeric data are expressed with means with standard deviation or median with range. Categorical data are shown as numbers with percentages. Independent sample 2-tailed *t* test with a significance level of .05 was used to compare means.

## Results

### Demographics

Twenty-one patients with fractures of the talar head were identified. The majority were male (n = 13, 62%), with an overall average age at the day of trauma of 40.5 (range, 18-74) years. More than two-thirds of patients were referred from other hospitals (n = 15, 71%). Reasons for referral included foot and ankle expertise (n = 11), accidents occurred abroad (n = 2), and delayed referral in case of missed fractures (n = 2). The mechanisms of trauma, in descending order of frequency, were fall during daily activities (n = 8), fall from height (n = 6), motor vehicle accident (MVA, n = 5), sport (n = 1), and blunt trauma (n = 1).

### Injury Pattern

**Figure 1.**
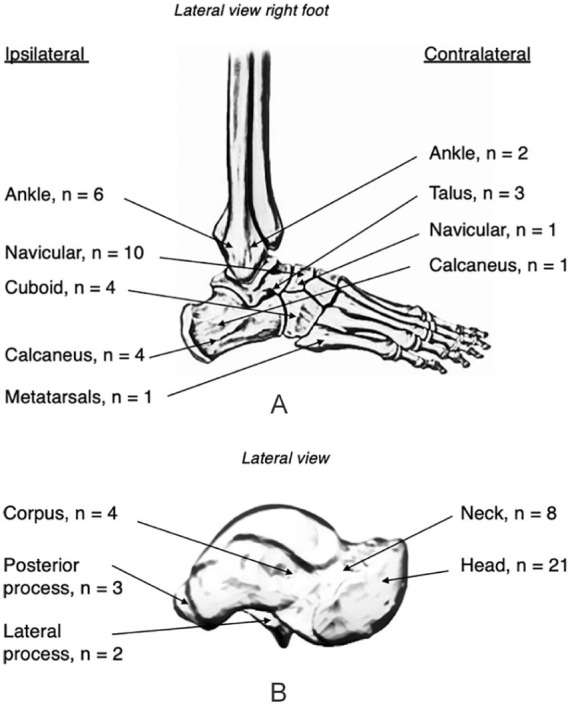
(A) Concomitant fractures. (B) Ipsilateral concomitant talus fractures.

Isolated talar head fractures were not encountered. Concomitant ipsilateral foot and/or ankle injuries other than the talus occurred in 71% of patients (n = 15) (Figure 1). The most common injury pattern was a fracture-dislocation (n = 13, 62%) at the talonavicular (n = 6), peritalar (n = 6), or talocrural (n = 1) joints. Half of our patients had a Chopart joint injury (n = 11). Two patients had additional Lisfranc injuries. Eight patients (38%) had a talar head fracture without other talus fractures, but all of them sustained other foot fractures. Four patients (19%) sustained fractures to the contralateral foot and ankle, of which 2 patients had bilateral talar fractures after a fall from height.

**Figure 2.**
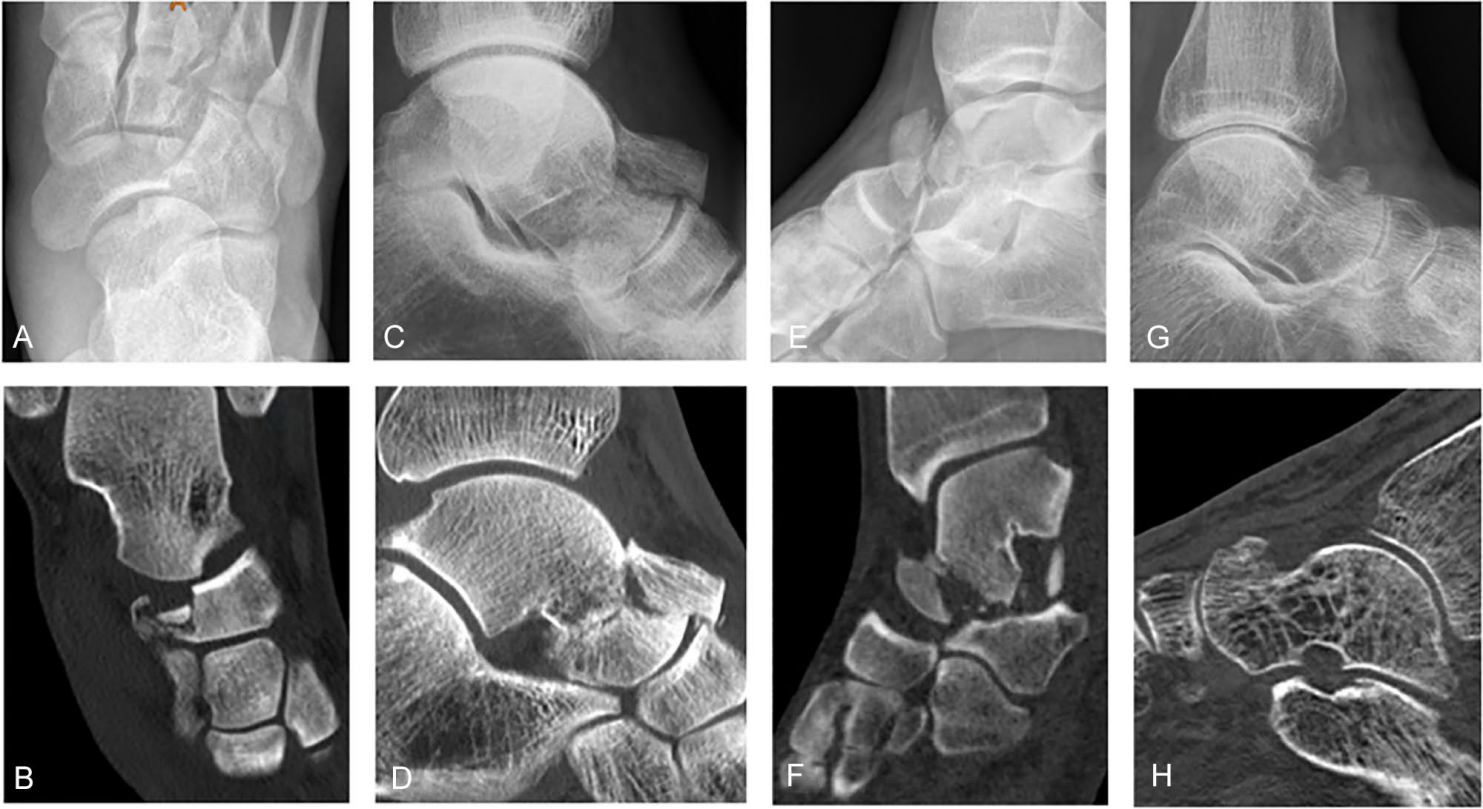
Four types of talar head fractures identified. Different fracture types of the talar head as shown in conventional radiographs (top row) and computed tomography scans (bottom row). (A, B) Impaction fracture of the talus head. (C, D) Complete transversal fracture of the talus head and fracture of the talus neck. (E, F) Medial shear fracture of the talus head, best seen in the sagittal plane. (G, H) Avulsion fracture of the talus head, first presented in our hospital at 4 months after trauma. Fracture was initially missed.

All patients were diagnosed using both conventional radiography and computed tomography (CT) imaging. Four types of fractures were identified: impaction (52%, n = 11), complete transverse (19%, n = 4), medial shear (14%, n = 3), and avulsion (14%, n = 3) fractures (Figure 2).

### Management

**Figure 3.**
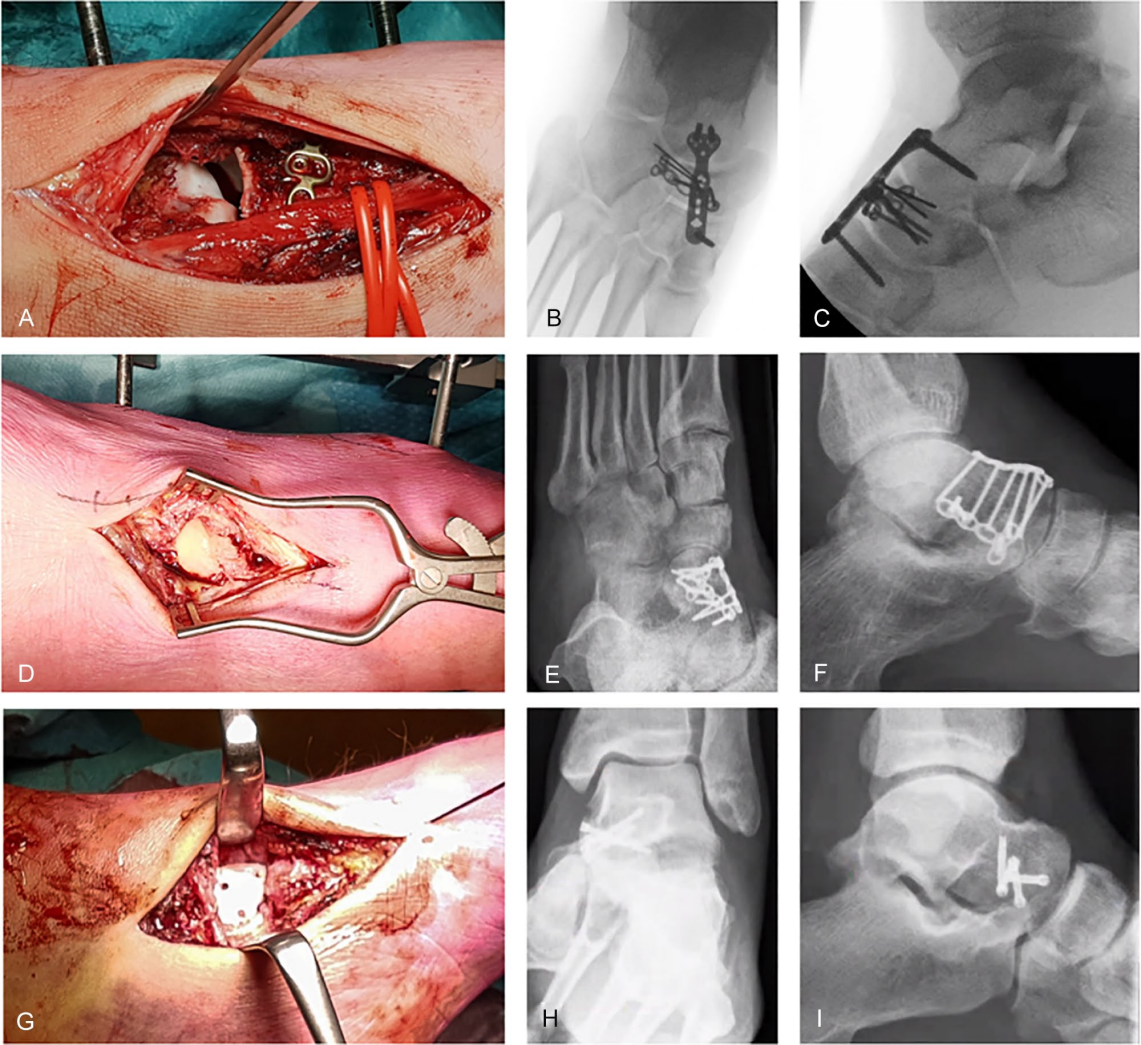
Examples of operative management. (A-C) Intraoperative images of an impaction fracture of the talus head with (A, B) concomitant comminuted lateral navicular fracture. Incision lateral of m. extensor hallucis longus (identified by vessel loops). Open reduction and internal fixation (ORIF) with 2.0 navicular plate and 2.7 bridge plate. (D-F) Complete transversal fracture of talus head with (C, D) associated neck fracture. Toes on the left side, dual approach via medial and anterolateral incisions. ORIF using 2 × 2.0 hand plate. (G-I) Medial shear fracture of the talar head as part of the (E, F) talonavicular fracture dislocation. ORIF using medial incision, 4× Headless Compression Screw 2.4 and K-wire due to talonavicular instability.

In total, 15 of 21 patients (71%) were treated operatively, of whom 13 underwent open reduction and internal fixation (ORIF) of the talar head fracture (Figure 3). One patient underwent ORIF with simultaneous primary talonavicular arthrodesis due to a locked fracture-dislocation with a comminuted navicular fracture and talar head impaction injury. The median period between the date of trauma and definitive surgery in the other patients (n = 14) was 8 (range, 0-15) days. Two patients received an external fixator as bridge to definitive surgery (13%). A single-incision approach on the anteromedial side was used in the majority of cases (n = 10). The other cases were managed via a dual approach (anteromedial and lateral or dorsomedial). In 5 of 15 operated patients, the implants were removed due to pain and/or functional impairment. Seventy percent of patients treated operatively returned to their normal daily activities, including work (n = 7/10 patients who returned the survey).

The remaining 6 talar head injuries were managed nonoperatively (29%) due to no or minimal displacement (n = 6, including 3 avulsions). Nevertheless, all patients in our nonoperative group underwent ORIF for associated ipsilateral talus, foot, or tibia fractures. Four of them had Chopart injuries, of whom 2 had fracture dislocations. Four of 6 nonoperatively managed patients who completed the survey returned to their normal daily activities (67%). The other 2 had complex bilateral (open) lower extremity fractures, including extensive soft tissue damage.

### Functional Outcome and Quality of Life

One patient died during follow-up due to a nonrelated cause. Follow-up time was insufficient in 1 patient. Questionnaires were sent to the remaining 19 patients, of whom 16 responded, yielding a total follow-up rate of 84%. Two patients could not be reached, and the other patient did not want to participate. The mean follow-up time from the day of trauma was 4.9 (range, 0.9-18) years.

Treatment characteristics, functional outcome, and patient-reported outcome measures per group are demonstrated in Table 2. Differences in functional outcome and quality of life between the operative and nonoperative group were not statistically significant. Out of the 4 fracture types, the lowest mean score was found in the group with shear fractures and the highest score in the patients with avulsion fractures (Table 3). There was a clear trend toward better functional outcome and quality of life in patients without a fracture dislocation and without ipsilateral talus or other foot or ankle fractures (Table 4).

**Table 2.** Treatment Characteristics, Functional Outcome, and Quality-of-Life Scores.^a^

Characteristic	Total (n = 21)	Operative (n = 15)	Nonoperative (n = 6)	*P* value
Available outcome data, n	16	10	6	
Functional outcome				
FFI, mean (SD)	34.2 (25.1)	38.0 (26.0)	28.0 (24.6)	.458
AOFAS, mean (SD)	70.7 (18.6)	70.5 (19.8)	71.0 (18.2)	.961
Excellent, n	3	2	1	
Good, n	4	3	1	
Fair, n	6	3	3	
Poor, n	3	2	1	
Quality of life, mean (SD)				
EQ-5D index	0.74 (0.18)	0.75 (0.18)	0.73 (0.2)	.810
Patient satisfaction	7.6 (1.8)	8.0 (1.8)	6.8 (1.7)	.217

Abbreviations: AOFAS, American Orthopaedic Foot & Ankle Society; EQ-5D, EuroQoL-5D; FFI, Foot Function Index.

a *P* values calculated with 2-tailed independent sample *t* test.

**Table 3.** Trends in Functional and Patient-Reported Outcome per Talar Head Injury Type.^a^

Characteristic	FFI	AOFAS	EQ-5D	Satisfaction
Type of fracture (n)				
Impaction (8)	24.2 (0-65.7)	72.5 (48-100)	75 (50-90)	8 (5-10)
Shear (3)	63.9 (44.3-66)	43 (36-58)	50 (40-70)	8 (6-9)
Avulsion (3)	22.2 (5.2-68.2)	82 (74-100)	90 (80-96)	9 (9-9)
Transverse (2)	34.6 (11.7-57.4)	74 (71-77)	90 (90-90)	5.5 (5-6)
Fracture dislocation (n)				
Present (10)	46.5 (1.3-65.7)	64.5 (36-90)	70 (40-90)	7.5 (5-10)
Absent (6)	17.0 (0-68.2)	78 (67-100)	90 (70-96)	9 (5-9)
Ipsilateral talus fractures (n)				
Present (11)	44.3 (1.3-68.2)	71 (36-100)	80 (40-96)	9 (5-10)
Absent (5)	22.2 (0-66)	81 (43-100)	80 (50-90)	7 (5-9)
Ipsilateral other foot/ankle fractures (n)				
Present (12)	31.4 (0-65.7)	68.5 (36-100)	70 (40-90)	7.5 (5-9)
Absent (4)	31.3 (1.3-68.2)	83.5 (74-100)	90 (90-96)	9 (5-10)

Abbreviations: AOFAS, American Orthopaedic Foot & Ankle Society; EQ-5D, EuroQoL-5D; FFI, Foot Function Index.

a Satisfaction refers to patient satisfaction (0 to 10). Data presented as median with range.

**Table 4.** Overview of Included Patients Who Have Hindfoot Trauma With Concomitant Talar Head Injury.

Patient n No.	Other ipsilateral lower extremity fractures	Type of talar head injury	AOFAS	FFI
1	Navicular	Impaction	100—excellent	0.0
2	Talus neck	Avulsion	100—excellent	5.2
3	Talonavicular luxation fracture, navicular	Impaction	90—excellent	1.3
4	Navicular, calcaneus	Avulsion	82—good	22.2
5	Navicular, cuboid	Impaction	81—good	21.3
6	Talus lateral process, talar luxation fracture	Transverse	77—good	57.4
7	Talus neck, posterior process talus, calcaneus, distal fibula and cuboid, Chopart luxation fracture	Impaction	75—good	48.7
8	Talus neck	Avulsion	74—fair	68.2
9	Distal tibia fracture, proximal fibula fracture, talus neck, navicular, calcaneus	Transverse	71—fair	11.7
10	Navicular, talus body, posterior talus, talonavicular luxation fracture	Impaction	70—fair	9.1
11	Navicular	Impaction	67—fair	27.0
12	Navicular, talus posterior process, talonavicular luxation fracture	Impaction	59—fair	35.7
13	Ankle luxation fracture, talus body, navicular, cuboid	Shear	58—fair	63.9
14	Lateral process talus, talus neck, open ankle luxation fracture	Impaction	48—poor	65.7
15	Distal fibula, talar/ankle luxation fracture	Shear	43—poor	66.0
16	Navicular, talonavicular luxation fracture	Shear	36—poor	44.3

Abbreviations: AOFAS, American Orthopaedic Foot & Ankle Society; FFI, Foot Function Index.

### Complications

No postoperative wound infections and no cases of avascular necrosis were observed. All fractures united, and 4 patients developed posttraumatic osteoarthritis (25%) at the talonavicular (TN, n = 3) and posterior talocalcaneal joint (PTC, n = 1). In total, 7 patients reported chronic foot pain (44%), including 4 patients in the operative group (including the patient with corrective osteotomy at 4 months after trauma) and 3 patients in the nonoperative group. Three patients managed operatively underwent secondary arthrodesis of the PTC joint (n = 2) or TN fusion (n = 1). All of them reported a decrease in pain afterward. No secondary arthrodesis was performed in the nonoperatively treated patients. Overall, the complication rate specifically linked to the talar head injury was 3 of 16 (19%).

## Discussion

Our series demonstrated that all talar head fractures coincided with other foot fractures, often in the context of peritalar fracture-dislocations or Chopart injuries. Diagnosis of a talar head fracture warrants careful examination and caution for a more complex injury. Conversely, when a fracture-dislocation or Chopart injury is observed, all contours of the talus should be carefully assessed for possible head fractures. This is supported by evidence on complex foot fractures from Rammelt et al.^16^ Involvement of the talar head was found in 14 of 61 patients with fractures at the Chopart joint. From the perspective of functional outcome and quality of life, patients with talar head fractures should be informed that their prognosis (of mostly fair to sometimes good long-term foot function) depends on the concomitant injuries (Table 3).

### Injury Mechanism

Four different groups of acute talar head fractures were identified in our cohort: impaction, complete transverse, medial shear, and avulsion fractures. Avulsion fractures are thought to be underreported in literature due to the larger, distracting injuries. It is thought that inversion motion through the talonavicular joint leads to a comminuted or shear fracture, resulting in medial column shortening and irreducible fracture-dislocation.^10^ Impaction injuries usually occur during high-energy trauma. Talar head fractures may also be the consequence of axial compression in full plantarflexion of the foot or, alternatively, hyperdorsiflexion as a result of impact against the anterior side of the tibia.^4,9,10^ An interesting finding is that impaction injury was both the most common type of fracture in our series and was mentioned specifically in previous reports of patients with talar head fractures.^7,9,22^ This may suggest that impaction is indeed a common cause of talar head fractures and that a high degree of suspicion of a talar head fracture should be raised in patients with impaction injuries to the foot, including talonavicular fracture dislocations. Early diagnosis of talar head fractures is crucial since this will influence articular congruity and thus management.

### Imaging

In conventional radiography, the head of the talus overlaps the calcaneus on the anteroposterior view.^14^ Therefore, CT should be performed to identify potentially subtle fractures, displacement, rotation, and/or extent into the neck or navicular bone.^12^

### Management

Given the broad range of possible associated injuries found, it is impossible to present a general surgical plan or provide detailed recommendations on how to reduce and fix every type of talar head fracture. In general, patients with more extensive fractures of the talar head with dislocation, impaction, and/or intra-articular gaps or step-offs were managed with ORIF. Based on our findings and the literature, avulsion fractures and other nondisplaced talar head fractures can be managed nonoperatively using a short leg cast or walking boot and nonweightbearing for approximately 4 weeks.^14^ Clinical and radiological (using CT) healing should be evident to rule out secondary rotation or displacement prior to weightbearing.^7,10,12,14^ In case of a larger fragment and/or in case of talonavicular joint instability, optimal management is ORIF to restore articular congruency.^2,7^ This also applies to smaller displaced intra-articular fragments from the talar head or navicular. Severely comminuted fractures with soft tissue damage were treated with temporary external fixation followed by ORIF.

In case of severe comminution of the talar head and/or navicular and/or destruction of the joint otherwise, primary fusion of the talonavicular joint may be required.^8,17^ Although preservation of the talonavicular joint should always be a major aim, secondary (salvage) arthrodesis is indicated for patients with persistent instability or pain due to posttraumatic osteoarthritis.

### Operative Technique

In general, a single incision was made on the anteromedial side for optimal reduction and fixation without extensive soft tissue damage.^1,2^ All transverse and medial shear fractures were approached in this way. As seen in our series and in the literature, a double-incision approach may be preferred in cases with impaction fractures (with concomitant navicular injuries) or if the fracture crosses the midline and extends to both sides of the talar head.^2,19^ The dual approach usually involves a direct anteromedial longitudinal incision over the tip of the medial malleolus, across the navicular tuberosity to the proximal aspect of the medial cuneiform (visualization and mobilization, screw fixation, spanning plate application), and a second incision laterally using the distal part of the sinus tarsi approach (reduction and fixation). We suggest that the approach should be tailored to the specific fracture type and associated injuries (Figure 2).

When reconstruction is required, displacement or shortening of the medial column can be managed using a small external fixator from the talar neck to the cuneiform or navicular to facilitate disimpaction and reduction. Stability of the medial column and talonavicular joint is critical, and the talonavicular joint capsule should be repaired after fixation. Internal fixation can be achieved with subchondral cancellous lag screws or bioabsorbable pins. Bridge plating with a locking plate may be used in case of joint instability.^2,7^ The role of percutaneous Kirschner wires is limited due to unstable fracture reduction and soft tissue complications.^1^ Fragment excision may be considered only in the rare case of an irreparable fracture.^2^ The usual postoperative protocol involves nonweightbearing for 2 to 8 weeks, followed by partial weightbearing in a walker and ankle mobilization exercises. Early exercises are allowed only if fixation is stable.

### Functional Outcome

In the literature, functional outcome of talar head fractures has been reported using a validated outcome only once before. Anderson^2^ showed that patients (n = 8) had decreased physical function and more pain compared to population norms. Regardless of the management type, about 70% of our patients returned to their normal daily activities during long-term follow-up. Mean FFI and AOFAS scores showed fair to good overall functional outcome, quality of life, and patient satisfaction. It should be noted that both patients treated operatively and nonoperatively sustained associated talus or other ipsilateral foot injuries. The group with poor outcome included the case of missed fracture, a patient who underwent secondary subtalar fusion and a patient with a severe Chopart injury. A trend toward a slightly better FFI in the nonoperative group was observed (28.0 vs 38.0), yet AOFAS scores were similar and patient satisfaction appeared to be higher in the operatively managed group (8.0 vs 6.8). Although multivariate analysis could not be performed due to small sample size, a trend toward lower functional outcome was observed in patients with shear fractures. In comparison to other studies regarding patients with different types of talar fractures, our AOFAS score (mean, 70.7) was found to be slightly lower than the outcome of central (mean, 78.9, 76.1, and 71.4), lateral (mean, 75.0), and posterior process (mean, 78.7) talar fractures.^3,18,23-25^ This supports our hypothesis that patients with (among others) talar head fractures usually face impaired functional outcome, interpreted as “fair” according to the AOFAS outcome measure. A possible explanation is that talar head fractures usually occur as part of more complex hindfoot trauma, as shown in this study. The finding that patients without a fracture dislocation and without ipsilateral talus or other foot or ankle fractures achieved higher functional outcome and quality-of-life scores was not surprising yet still relevant in clinical practice. Shared decision making and expectation management are essential and may lead to high patient satisfaction despite impaired functional outcome.

### Complications

About a quarter of patients developed posttraumatic osteoarthritis in our series, compared to 50% reported in literature.^2^ Operative complications were not found or reported in the literature. In particular, the incidence of avascular necrosis was found to be zero in both the literature and our series. This is probably due to the abundant blood supply to the talar head by the periosteal branches of the dorsal pedis artery and tarsal sinus artery that runs anteriorly.^2,7^

### Limitations

This is the largest series of acute talar head fractures with validated outcome measures. Due to the very low incidence of this fracture, the sample size was small. No patients with isolated talar head fractures were found. Various types of talar head fractures were recognized, yet no conclusions on the difference between their functional outcome and prognosis could be drawn. Minimally displaced or avulsion fractures were managed well nonoperatively. Others should be treated with ORIF. Another limitation is the high incidence of concomitant injuries, which likely biased the functional outcome and quality of life (Table 3).

## Conclusion

Talar head fractures always coincided with other (foot) fractures. Next to the talonavicular joint, other Chopart joint articulations are at risk of injury when a talar head fracture is diagnosed. Both the medial and lateral column should therefore be carefully assessed for injury. Management and long-term functional outcome were affected by the extent of associated injuries. Due to the low incidence and high complexity of talar head fractures, early referral to dedicated foot surgeons and centralization of complex foot surgery are recommended.

## Supplemental Material

sj-jpg-1-fai-10.1177_1071100720980023 – Supplemental material for Management and Outcome of Hindfoot Trauma With Concomitant Talar Head Injury Click here for additional data file. Supplemental material, sj-jpg-1-fai-10.1177_1071100720980023 for Management and Outcome of Hindfoot Trauma With Concomitant Talar Head Injury by Esmee Wilhelmina Maria Engelmann, Olivier Wijers, Jelle Posthuma and Tim Schepers in Foot & Ankle International

sj-pdf-1-fai-10.1177_1071100720980023 – Supplemental material for Management and Outcome of Hindfoot Trauma With Concomitant Talar Head Injury Click here for additional data file. Supplemental material, sj-pdf-1-fai-10.1177_1071100720980023 for Management and Outcome of Hindfoot Trauma With Concomitant Talar Head Injury by Esmee Wilhelmina Maria Engelmann, Olivier Wijers, Jelle Posthuma and Tim Schepers in Foot & Ankle International

sj-pdf-2-fai-10.1177_1071100720980023 – Supplemental material for Management and Outcome of Hindfoot Trauma With Concomitant Talar Head Injury Click here for additional data file. Supplemental material, sj-pdf-2-fai-10.1177_1071100720980023 for Management and Outcome of Hindfoot Trauma With Concomitant Talar Head Injury by Esmee Wilhelmina Maria Engelmann, Olivier Wijers, Jelle Posthuma and Tim Schepers in Foot & Ankle International
