# Supplementary figures and images for: Management and Outcome of Hindfoot Trauma With Concomitant Talar Head Injury
Source: Foot Ankle Int. 2021 Jan 21;42(6):714–22. doi: 10.1177/1071100720980023 (PMC8209765; doi:10.1177/1071100720980023)

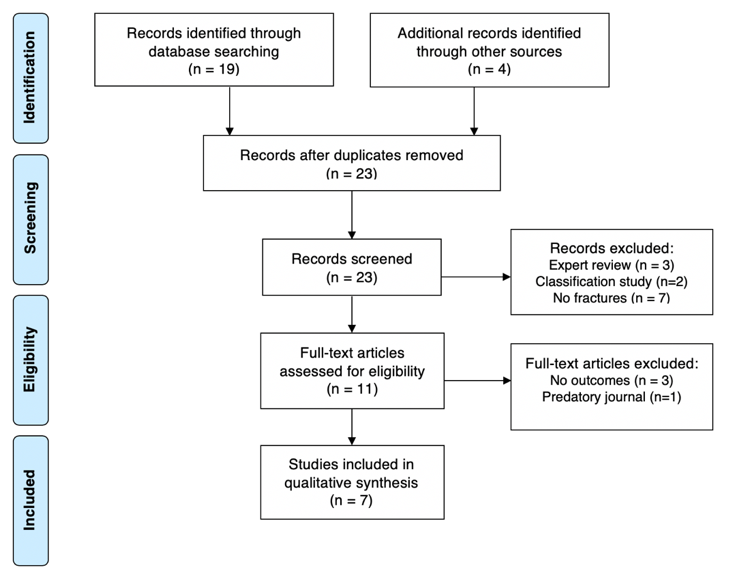

Supplement: sj-jpg-1-fai-10.1177_1071100720980023 – Supplemental material for Management and Outcome of Hindfoot Trauma With Concomitant Talar Head Injury [file sj-jpg-1-fai-10.1177_1071100720980023.jpg]
