# Supplementary material for: Management and Outcome of Hindfoot Trauma With Concomitant Talar Head Injury
Source: Foot Ankle Int. 2021 Jan 21;42(6):714–22. doi: 10.1177/1071100720980023 (PMC8209765; doi:10.1177/1071100720980023)
Supplement: sj-pdf-2-fai-10.1177_1071100720980023 – Supplemental material for Management and Outcome of Hindfoot Trauma With Concomitant Talar Head Injury [file sj-pdf-2-fai-10.1177_1071100720980023.pdf]

Table 4. Overview of included patients suffering hindfoot trauma with concomitant talar head injury

Patient nr	Other ipsilateral lower extremity fractures	Type of talar head injury	AOFAS	FFI
1	Navicular	Impaction	100 - EXCELLENT	0.0
2	Talus neck	Avulsion	100 - EXCELLENT	5.2
3	TN luxation fracture, navicular	Impaction	90 - EXCELLENT	1.3
4	Navicular, calcaneus	Avulsion	82 - GOOD	22.2
5	Navicular, cuboid	Impaction	81 - GOOD	21.3
6	Talus lateral process, talar luxation fracture	Transverse	77 - GOOD	57.4
7	Talus neck, posterior process talus, calcaneus, distal fibula and cuboid, Chopart luxation fracture	Impaction	75 - GOOD	48.7
8	Talus neck	Avulsion	74 - FAIR	68.2
9	Distal tibia fracture, proximal fibula fracture, talus neck, navicular, calcaneus	Transverse	71 - FAIR	11.7
10	Navicular, talus body, posterior talus, talonavicular luxation fracture	Impaction	70 - FAIR	9.1
11	Navicular	Impaction	67 - FAIR	27.0
12	Navicular, talus posterior process, talonavicular luxation fracture	Impaction	59 - FAIR	35.7
13	Ankle luxation fracture, talus body, navicular, cuboid	Shear	58 - FAIR	63.9
14	Lateral process talus, talus neck, open ankle luxation fracture	Impaction	48 - POOR	65.7
15	Distal fibula, talar/ankle luxation fracture	Shear	43 - POOR	66.0
16	Navicular, talonavicular luxation fracture	Shear	36 - POOR	44.3
